# Modulation of Orosomucoid-like Protein 3 Activity in the Management of Inflammatory Bowel Disease

**DOI:** 10.26502/jbb.2642-91280167

**Published:** 2024-10-18

**Authors:** Ugljesa Malicevic, Vikrant Rai, Ranko Skrbic, Devendra K Agrawal

**Affiliations:** 1 Department of Translational Research, Western University of Health Sciences, Pomona, California 91766, USA; 2 Centre for Biomedical Research, Faculty of Medicine, University of Banja Luka, Banja Luka, Republic of Srpska, Bosnia and Herzegovina, Balkans; 3 Departments of Pathophysiology, Pharmacology, Toxicology and Clinical Pharmacology, Faculty of Medicine, University of Banja Luka, Banja Luka, Republic of Srpska, Bosnia and Herzegovina, Balkans

**Keywords:** Autophagy, Crohn’s disease, Inflammation, Inflammatory bowel disease, Mitochondrial dysfunction, Network analysis, NLRP3, ORMDL3, Sphingolipid biosynthesis regulator, Therapeutic targets, Toll-like receptors, Ulcerative colitis

## Abstract

Inflammatory bowel disease (IBD), encompassing Crohn’s disease and ulcerative colitis, is a chronic and relapsing condition characterized by persistent inflammation of the gastrointestinal tract. The complex pathogenesis of IBD involves a combination of genetic, environmental, and immune factors, which complicates the achievement of long-term remission. Lower abdominal pain, stomach cramps, blood in stool, chronic diarrhea, fatigue, and unexpected weight loss are common presenting symptoms. Despite the range of therapies and medications, including anti-inflammatory and anti-diarrheal drugs, immunosuppressants, antibiotics, and analgesics aimed at managing symptoms and controlling inflammation, a definitive cure for IBD remains elusive. Current therapy targets inflammation, mainly cytokines, inflammatory receptors, and immune cells, however, there is a need for novel targets to improve clinical outcomes. To identify novel targets and interactions among various factors, we performed a network analysis using various cytokines, TLRs, and NLRP3 inflammasome as inputs. This analysis revealed orosomucoid-like protein 3/ORMDL sphingolipid biosynthesis regulator 3 (ORMDL3) as a central hub gene interacting with multiple factors. While the role of ORMDL3 in IBD pathogenesis is not well-established, our findings and existing literature suggest that ORMDL3 plays a role in inflammation, impaired mitochondrial function, and disrupted autophagy, all contributing to the disease progression. Given its central role in these pathogenic processes, targeting ORMDL3 presents a promising therapeutic target. Modulating ORMDL3 activity could alleviate inflammation, restore mitochondrial function, and enhance autophagy, potentially leading to more effective treatments and improved outcomes for IBD patients.

## Introduction

**Figure 1:**
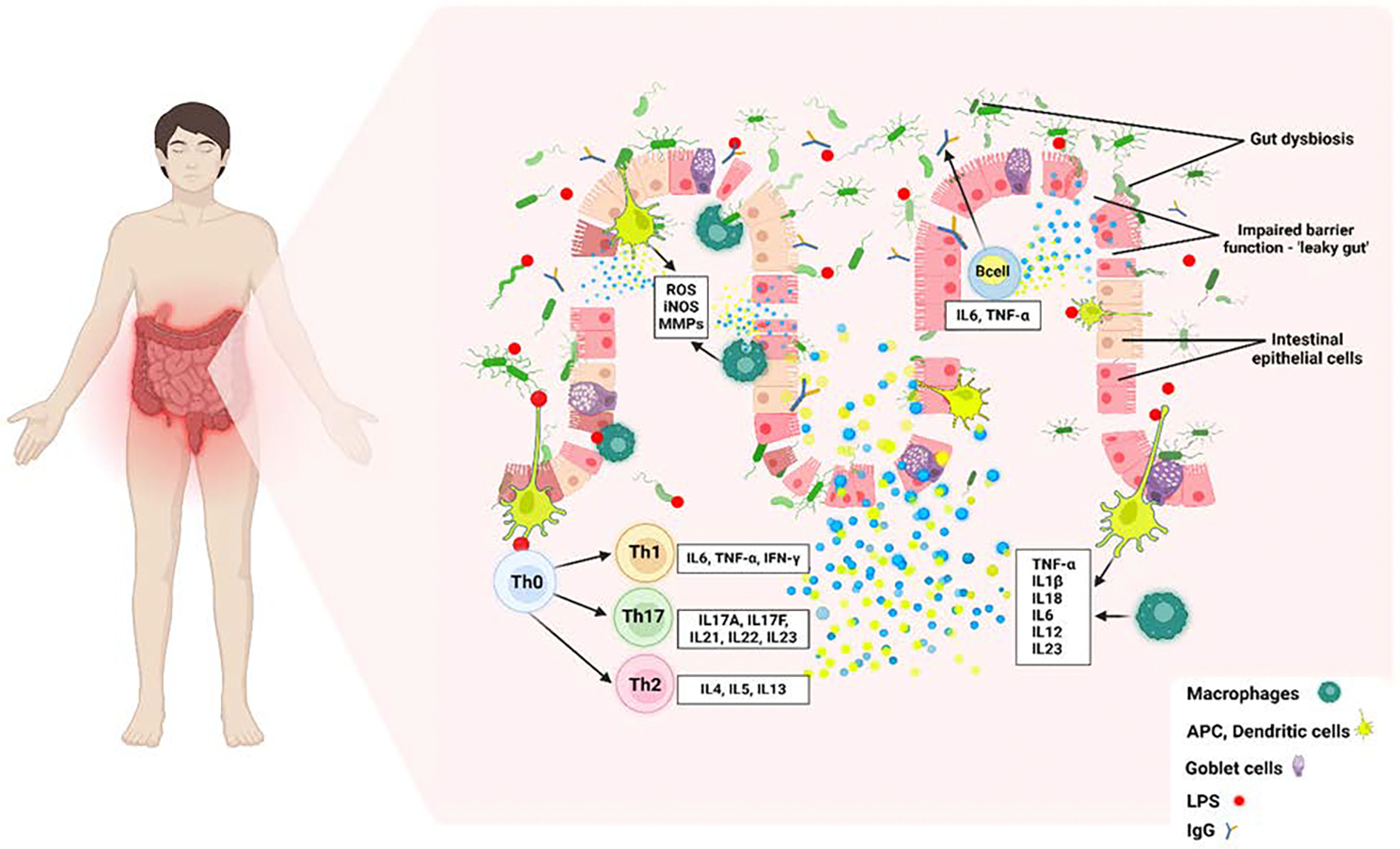
Cytokines involved in the pathogenesis of inflammatory bowel disease (IBD). This figure highlights the key pro-inflammatory cytokines involved in the pathogenesis of IBD, such as TNF-α, IL-1β, IL-6, IL-17, and interferon (IFN)-γ. These cytokines contribute to initiating and perpetuating inflammation within the intestinal mucosa, leading to tissue damage, leaky intestinal barrier, and the chronic inflammatory state characteristics of conditions such as Crohn’s disease and ulcerative colitis. Dysregulated production and signaling of these cytokines play a central role in driving the immune response in IBD. The compositional and metabolic changes in the intestinal microbiota (dysbiosis) also play a critical role in the pathogenesis of IBD. APC, antigen presenting cells; iNOS, induced nitric oxide synthase; LPS, lipopolysaccharide; MMPs, matrix metalloproteinases; ROS, reactive oxygen species.

Inflammatory bowel disease (IBD) is a long-lasting, progressive, recurrent, and chronic disease primarily affecting the digestive system, most often the small intestine and colon [1]. The main types of IBD include ulcerative colitis (UC) and Crohn’s disease (CD). Although their symptoms have many similarities, these disorders are different entities with various etiologies, including genetic, environmental, and immunologic factors [2]. UC primarily affects the colon and rectum, leading to surface mucosal inflammation and often resulting in erosions and ulcers. In contrast, Crohn’s disease may affect any part of the digestive system and is characterized by transmural inflammation, leading to strictures, fistulas, and abscesses [3]. Individuals diagnosed with IBD commonly suffer from various gastrointestinal symptoms, including persistent diarrhea, abdominal pain, cramping, and bloody stool. Furthermore, they may also experience non-gastrointestinal symptoms such as fatigue, depression, anxiety, weight loss, etc. [4,2]. IBD primarily affects young adults, with over 25% of patients exhibiting symptoms before turning 20 years old [5]. Nonetheless, it is important to note that this disease can develop at any age. Most commonly individuals between the ages of 20 and 40 years are diagnosed with IBD, which can significantly impact all aspects of a patient’s life [6]. Furthermore, there is evidence of a bimodal distribution, suggesting the existence of a second peak at the age of 60, when about 10% to 15% of individuals develop this disease [7]. North America and Northwestern Europe recorded the highest prevalence of IBD in the 20th century, primarily affecting the white population, especially Ashkenazi Jews. Conversely, the incidence of IBD in past years has increased significantly in newly industrialized and developing countries across Asia, Latin America, South America, and Africa, establishing this condition as a global problem [8,9]. IBD is primarily treated pharmacologically, using corticosteroids, aminosalicylates, immunomodulators, and biologics as standard treatments to control symptoms [1]. Because cytokines play a crucial role in IBD development, various therapeutic strategies are used to regulate and target inflammation, providing symptom relief. Key treatment targets include Tumor Necrosis Factor-alpha (TNF-α), which is inhibited by drugs such as infliximab, adalimumab, and certolizumab pegol to block its activity. Ustekinumab targets interleukin (IL)-12 and IL-12/23, while vedolizumab targets integrins to prevent immune cell migration to the gut and cause inflammation. Janus Kinase (JAK) inhibitors, such as tofacitinib, block one or more JAK enzymes to disrupt cytokine signaling. Additionally, sphingosine-1-phosphate (S1P) receptor modulators, like ozanimod, reduce lymphocyte migration. Experimental treatments are also being explored to target several cytokines, including IL-1, IL-6, IL-17, IL-18, and IL-22. These treatments aim to block or modify the cytokines to control inflammation more effectively and help the intestinal mucosa repair [10–12] (Figure 1).

Despite major advancements in treating this disease, the present therapy is still not sufficiently effective in achieving long-term remission, reducing symptoms, and improving the quality of life for patients. Considering that chronic inflammation is the leading problem in this disease, targeting the genes involved and contributing to chronic inflammation is essential for developing newer treatment options for IBD [13] to improve clinical outcomes. The immune response plays a crucial role during inflammation, and the orosomucoid-like protein 3/ORMDL sphingolipid biosynthesis regulator 3 (*ORMDL3*) gene has been linked to increased inflammation in IBD. This gene regulates IL-1β secretion and immune cell activity, possibly through its effects on sphingolipid and calcium metabolism, as well as related inflammatory pathways [14]. However, the association of ORMDL3 with IBD and its potential as a therapeutic target are scarcely discussed. This article provides a comprehensive overview on the role of ORMDL3 in IBD pathogenesis, followed by the potential of targeting this molecule in the treatment of IBD.

### *ORMDL3* gene and biological function

**Figure 2:**
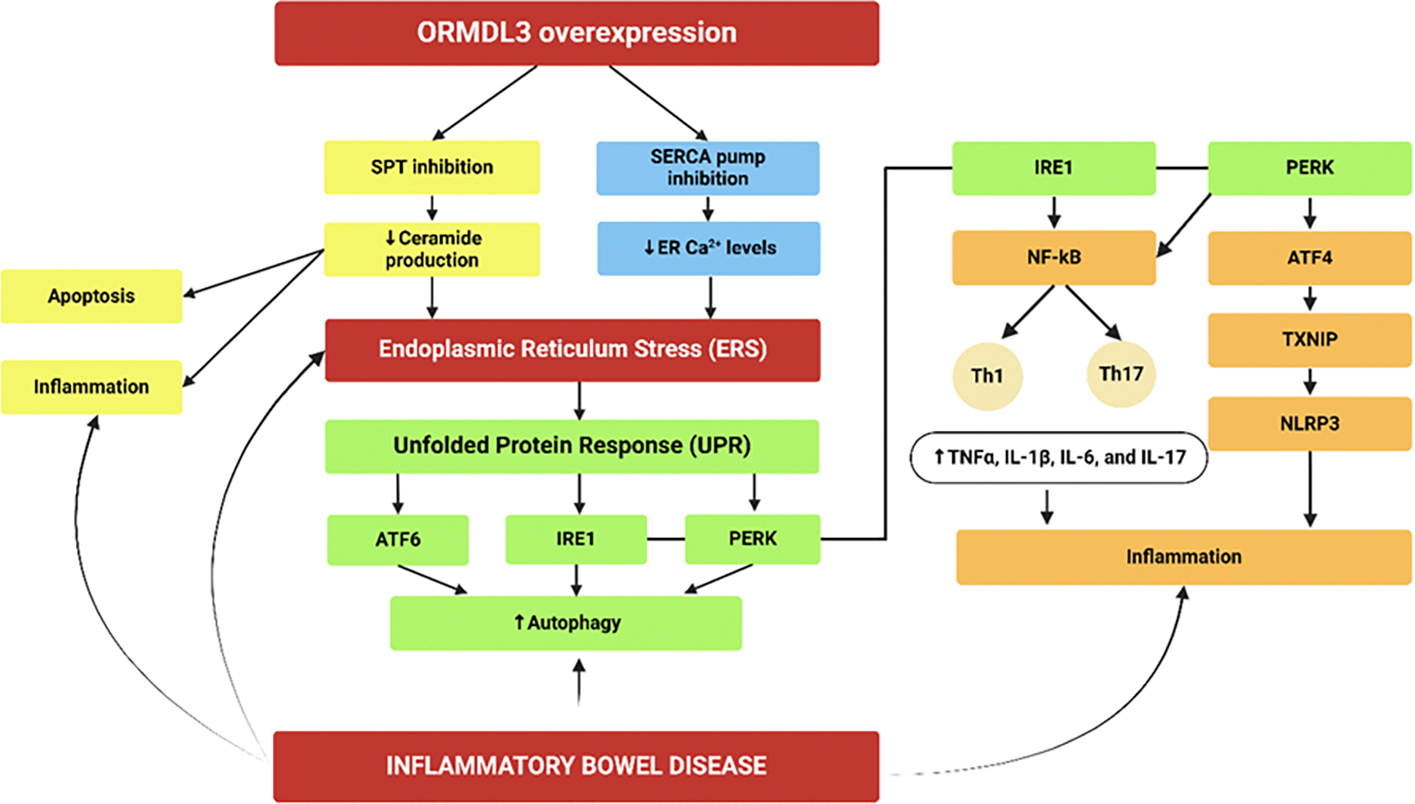
The proposed pathogenic role of ORMDL3 overexpression in inflammatory bowel disease (IBD) through modulation of its biological functions. Increased apoptosis, inflammation, autophagy, oxidative stress, endoplasmic reticulum stress, and activation of NLRP3 inflammasome are involved in the pathogenesis of IBD. The association of all these aspects with ORMDL3 overexpression suggests that ORMDL3 may play a critical pathologic role and may be a potential therapeutic target. ATF, activating transcription factor; IRE1, inositol-requiring enzyme 1; PERK, protein kinase R like ER kinase; PKR, protein kinase R; SERCA, sarco-endoplasmic reticulum Ca^2+^ ATPase; SPT, serine palmitoyl transferase; TXNIP, thioredoxin-interacting protein; NLRP3, NOD-, LRR- and pyrin domain-containing protein 3.

In 2002, Hjelmqvist et al. discovered the unique ORMDL human gene family, which includes the transmembrane proteins ORMDL1, ORMDL2, and ORMDL3 [15]. These proteins are present in the endoplasmic reticulum (ER), with ORMDL3 being the most well-known and extensively studied. The ORMDL Sphingolipid Biosynthesis Regulator 3 (*ORMDL3*) gene, found on human chromosome 17q21, consists of five exons and encodes a 153-amino acid protein anchored to the ER [16]. ORMDL3 and ORMDL2 are most highly expressed in myocytes (muscle cells), immune cells, and epithelial cells. In contrast, ORMDL1 is more abundantly expressed in various tissues, including the heart, brain, lung, liver, skeletal muscle, and kidney [17]. Initially, the function of the ORMDL proteins was unclear after their discovery [18]. However, subsequent research has revealed their involvement in a range of cellular processes. Maintaining homeostatic calcium ion (Ca^2+^) levels within the ER is essential for creating an optimal environment for proper protein folding. Any imbalance in these levels can lead to increased ER stress, unfolded protein response (UPR) activation, and various pathological conditions [19]. The UPR in the ER is an important adaptive response that keeps protein folding and translation in balance, controls the immune system’s response to various stimuli like allergens and infections, and acts as a “fight or flight” response [20]. Calcium ions are released from the ER into the cytoplasm through Ca^2+^ release channels and are subsequently returned to the ER by sarco-endoplasmic reticulum Ca^2+^ ATPase (SERCA) pumps. The activity of these pumps controls the rate of Ca^2+^ removal from the cytoplasm, crucially shaping calcium signaling and maintaining low cytosolic Ca^2+^ levels [21]. ORMDL3 has been found to be a regulator of the SERCA pump, and its overexpression disrupts calcium homeostasis by impairing Ca^2+^ uptake into the ER. This disruption further contributes to ER stress and triggers the UPR [22]. ORMDL3 directly interacts with and inhibits the SERCA pump, thereby preventing the transport of cytoplasmic calcium back into the ER. This inhibition is proven to increase protein kinase R (PKR) like ER kinase-eukaryotic initiation factor-2 alpha (PERK-eIF2α) signaling and heightened UPR activity in human embryonic kidney cells [23]. Decreased ER calcium levels initiate the UPR and involve the activation of signaling molecules and increased transcriptional activity of immediate-early genes and other genes associated with inflammation onset [24] (Figure 2).

In the ER, the ORMDL3 gene encodes a transmembrane protein that regulates the activity of serine palmitoyl transferase (SPT), an enzyme crucial for sphingolipid synthesis in cells [25]. Sphingolipids are bioactive molecules essential for cellular signaling and the creation and maintenance of cellular membrane integrity. They regulate various cellular processes, including cell growth, differentiation, apoptosis, and immune responses [26,27]. Researchers initially discovered that ORMDL3 regulates the de novo production of sphingolipids by negatively regulating SPT activity. This regulation begins in the ER, where serine and palmitoyl coenzyme A (CoA) combine via the enzyme SPT to form 3-ketosphinganine, the rate-limiting step in sphingolipid biosynthesis. Subsequent reactions convert 3-ketosphinganine into ceramide, which is further transformed into sphingomyelin and glycosphingolipids [28]. ORMDL3 overexpression inhibits sphingolipid synthesis by reducing SPT activity, leading to decreased ceramide production. This reduction in ceramide levels can disrupt various cellular processes, potentially causing increased apoptosis, inflammation, and even tumorigenesis [29] (Figure 2). The disturbance in sphingolipid metabolism underscores the critical role of maintaining balanced ceramide levels for cellular homeostasis and preventing disease development. As a result, the role of ORMDL3 in regulating SPT activity highlights the importance of maintaining cellular health and the potential implications for treatment strategies that target sphingolipid pathways in different diseases.

ORMDL3 is classified as an autophagy-associated gene and impacts autophagy by regulating sphingolipid metabolism, involvement in ERS and UPR, maintenance of calcium homeostasis, and modulation of the inflammatory response [30]. UPR consists of three main signaling pathways that are triggered by the activation of three protein sensors: PKR-like ER kinase (PERK), inositol-requiring enzyme 1 (IRE1), and activating transcription factor 6 (ATF6). The three branches of the UPR collectively control the process of autophagy [31] (Figure 2). Autophagy, crucial for cellular survival and homeostasis, is vital in numerous prevalent diseases, infections, cancers, and degenerative and autoimmune disorders [32,16]. Recent findings suggest that ORMDL3 may mediate autophagy through the ATF6 branch of the UPR, which is proposed to be essential for inducing autophagy. ORMDL3 activates the ATF6 pathway, increasing Beclin-1 transcription, a gene involved in autophagy [33]. Interferon-gamma (IFN-γ) triggers the cleavage of ATF6 and the ERK1/2-dependent phosphorylation of C/EBP-β. This process leads to the expression of death-associated protein kinase 1 (DAPK1), a Ca^2+^/calmodulin-regulated serine/threonine kinase. DAPK1 promotes the formation of autophagosomes by phosphorylating Beclin-1 [34]. Thus, the induction of autophagy is linked to the activation of the ATF6 pathway and the downstream signaling events involving DAPK1. ORMDL3 stimulates the ATF6 pathway, increasing autophagic activity in mast cells. When activated by an antigen, mast cells release various pro-inflammatory cytokines and chemokines, making them essential effector cells in IgE-dependent allergic disorders. ORMDL3 overexpression inhibits mast cell degranulation, reduces the production of pro-inflammatory cytokines and chemokines, and increases mast cell autophagic activity, suggesting that the ATF6 UPR-autophagy pathway is critical in controlling this process [33].

ORMDL3 has been implicated in activating several inflammatory pathways, including the modulation of the nuclear factor-kappa B (NF-κB) pathway, a crucial regulator of inflammation. As previously discussed, elevated ORMDL3 levels can induce ERS, which in turn activates the UPR. The UPR, particularly through its IRE1 branch, may increase the expression of inflammatory genes regulated by NF-κB. This ORMDL3-mediated activation of NF-κB significantly influences T-cell differentiation, promoting the development of pro-inflammatory Th17 and Th1 cells within the cytokine environment [35] (Figure 2). This regulation profoundly impacts the immune response, contributing to various inflammatory and autoimmune diseases such as IBD, asthma, rheumatoid arthritis, and multiple sclerosis. It is also noteworthy that the PERK branch of the UPR promotes the expression of thioredoxin-interacting protein (TXNIP) via activating transcription factor 4 (ATF4), which subsequently activates the NOD-, LRR- and pyrin domain-containing protein 3 (NLRP3) inflammasome, further amplifying inflammation [36]. Additionally, ER stress caused by disturbances in calcium levels can activate the stimulator of the interferon genes (STING) pathway, leading to the production of interferon-1 (IFN-1) [37]. Activation of the STING pathway and subsequent interferon-1 (IFN-1) production can have significant effects. While IFN-1 plays a crucial role in driving protective immune responses and enhancing antiviral defenses, its activation in chronic inflammation can also contribute to disease progression and the development of autoimmunity [38] (Figure 2).

### ORMDL3 interacts with mediators involved in IBD pathogenesis

**Figure 3:**
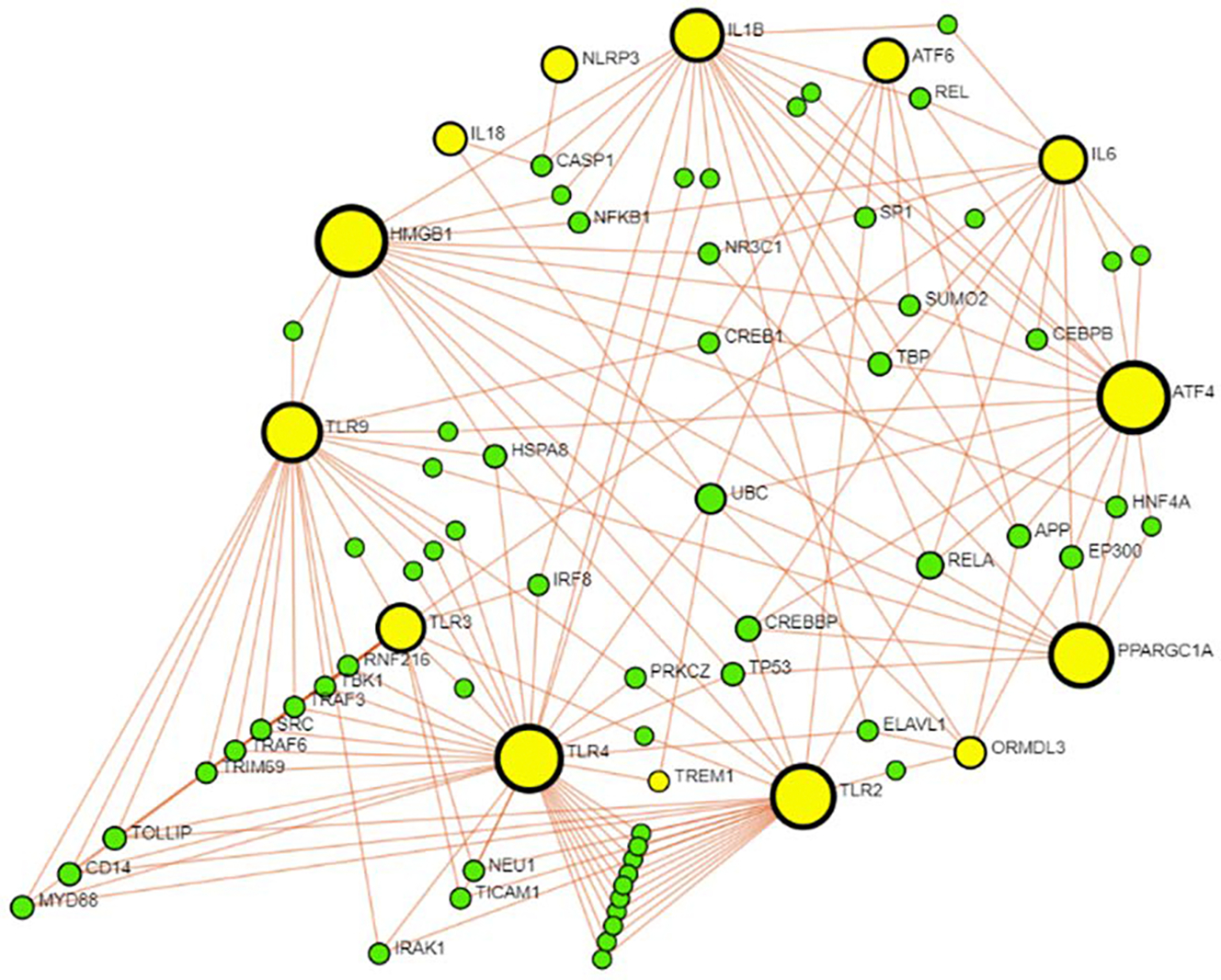
Protein-protein interaction between various mediators of inflammation, autophagy, and mitochondrial biogenesis via network analysis using NetworkAnalyst.ca. Network analysis conducted to delineate the interaction of various proteins (encoded by genes) involved in IBD pathogenesis with ORMDL3 revealed the interaction of ORMDL3 with various proteins whose altered expression is associated with IBD onset and progression. The interaction between various proteins and ORMDL3 suggests ORMDL3 as a potential therapeutic target in IBD treatment. For abbreviation of various proteins, please see the description in the text.

IBD is indeed characterized by chronic and persistent inflammation, with dysbiosis being considered one of the main factors contributing to inflammation [39]. An impaired intestinal barrier enables bacteria, metabolites, and toxins to directly interact with the intestinal epithelium, resulting in the infiltration of immune cells, increased production of pro-inflammatory cytokines, oxidative stress, and the release of lipopolysaccharide (LPS). Toll-like receptors (TLRs), particularly TLR2, TLR3, TLR4, and TLR9, and NOD-like receptors (NLRs) such as NLRP3, play a crucial role in recognizing microbial components and other pathogen-associated molecular patterns (PAMPs) and damage-associated molecular patterns (DAMPs) like HMGB1, possibly through heat shock protein A8 (HSPA8) (Figure 3). Along with these mediators, pro-inflammatory cytokines, including IL-1, IL-6, TNF-α, IL-18, IL-12, and IL-22, play a critical role in the pathogenesis of IBD [40,41,42] (Figures 1 and 2). ORMDL3 gene is overexpressed in many inflammatory diseases, including IBD, and plays a significant role in inflammation. However, the exact role of ORMDL3 in IBD pathogenesis and its interactions with the other mediators discussed in the previous paragraph is not well understood. Also, the regulatory networks, involved pathways, and the effects of protein-protein interactions and their role in IBD pathogenesis are still unknown. To predict various protein-protein interactions (PPI), we performed network analysis (https://www.networkanalyst.ca) using IMEx database with ORMDL3, TLR2, TLR3, TLR4, TLR9, IL6, IL1β, IL18, IL22, IL12, NLRP3, high mobility group box 1 (HMGB1), TNF-α, CASPASE1, CASPASE 3, CASPASE8, ATF4, ATF6, PPAR-γ coactivator-1 alpha (PGC-1α), receptor for advanced glycation end-products (RAGE), triggering receptor expressed on myeloid cells 1 (TREM1), NF-κB, JNK, and mitogen-activated protein kinases (MAPK) as input. The network analysis revealed a direct interaction between ORMDL3, TLR2, TLR4, TLR9, HMGB1, IL1β, IL18, ATF4, ATF6, and PGC-1α (Figure 3), suggesting a probable role of ORMDL3 in the pathogenesis of IBD. Further, the findings from the analysis indicated the interaction of TLRs and cytokines involved in IBD pathogenesis with ORMDL3, autophagy markers (ATF4 and ATF6), and mitochondrial biogenesis (PGC-1α) in the pathogenesis of IBD.

Network analysis suggests ubiquitin C (UBC) as a central hub connecting various proteins involved in inflammatory and immune responses, including ORMDL3, TLR4, TREM1, HMGB1, and IL18, all amplifying inflammation in various inflammatory diseases by triggering the release of pro-inflammatory cytokines [43,44,45,46]. Various stress conditions, such as heat shock, oxidative stress, and inflammatory signals, can induce the expression of UBC, indicating its importance in the cellular stress response [47]. In the context of IBD, TLR4 plays a pivotal role, and its interaction with UBC underscores that role in the pathogenesis of the disease. Alterations in gut microbiota significantly contribute to IBD, where TLR4 serves as a key pattern recognition receptor (PRR) for LPS from Gram-negative bacteria. Upon LPS binding, TLR4 activates two major signaling pathways: the MyD88-dependent and MyD88-independent pathways. In the MyD88-dependent pathway, MyD88 recruits and activates IL-1 receptor-associated kinase-4 (IRAK-4) and IL-1 receptor-associated kinase-1 (IRAK-1), which subsequently leads to the activation of TNF receptor-associated factor 6 (TRAF6). TRAF6 interacts with the ubiquitin-conjugating enzyme UBC13 and its cofactor UEV1A to form a complex, triggering the activation of transforming growth factor-β-activated kinase 1 (TAK1). Activated TAK1 transmits signals through several downstream pathways, including the IKK complex and the mitogen-activated protein kinase (MAPK) pathways. This signaling cascade leads to the activation of transcription factors such as AP-1, and NF-κB, which subsequently induce the production of pro-inflammatory cytokines such as IL-6, IL-1, and TNF-α [48,49]. Activation of all these mediators could potentially affect ORMDL3 gene overexpression, further contributing to inflammation.

Ubiquitination plays a fundamental role in regulating both MyD88-dependent and MyD88-independent TLR4 signaling pathways, highlighting its importance in controlling inflammation and immune responses in IBD (Figure 3). High mobility group box 1 (HMGB1) is another essential player in this inflammatory network. As a DAMP molecule, HMGB1 can be released actively or passively and engages in immunological responses by binding to various receptors, including TLR-4, -2, and -9. This binding increases the production of downstream inflammatory cytokines, creating a positive feedback loop that intensifies the inflammatory response in the intestines [50,51]. UBC-mediated ubiquitination processes can modulate the stability and function of proteins involved in immune responses, including those responding to extracellular HMGB1 [52]. Dysregulation of these processes can lead to prolonged activation of NF-κB, exacerbating inflammation by allowing HMGB1 to continue activating immune responses as a DAMP molecule. Both UBC and HMGB1 are integral to maintaining a healthy intestinal barrier as well. UBC influences the stability of proteins associated with the barrier, while HMGB1 can modulate inflammation and consequently affect barrier integrity [53,54] (Figure 3). Dysregulation of ubiquitin-mediated processes and elevated levels of HMGB1 can compromise tight junction integrity [55]. When these disturbances occur within the gastrointestinal tract, they lead to impaired barrier function, commonly known as “leaky gut.” This compromised intestinal epithelial barrier contributes significantly to the development and progression of various gastrointestinal disorders, including IBD. Impaired calcium levels can affect the intestinal barrier as well. Namely, calcium ions play a critical role in the function of cadherins, which are involved in cell-cell adhesion and tight junction formation [56]. Disruptions in calcium levels can destabilize tight junctions, increasing intestinal permeability. ORMDL3, when upregulated, inhibits the SERCA pump, reducing calcium uptake into the ER. This results in increased cytosolic and decreased ER calcium levels, compromising tight junction integrity. Elevated cytosolic calcium and resulting ER stress can further promote the secretion of pro-inflammatory cytokines, exacerbating inflammation in the gut mucosa [57]. Conversely, reduced ER calcium levels can induce ER stress, triggering UPR [58].

NLRP3 is another crucial PRR activated by pathogen-associated molecular proteins (PAMPs) and DAMPs, and its activation significantly contributes to IBD pathogenesis. This inflammasome plays a significant role in activating pro-inflammatory cytokines, particularly IL-1β and IL-18, through the activation of caspase-1 (Figure 3). These are the main cytokines in the pathogenesis of IBD, and although, according to network analysis, NLRP3 has a direct interaction only with them, it is also important to emphasize that NLRP3 is a component of a larger network of inflammatory responses that can lead to intestinal injury and mucosal erosion. The NLRP3 can indirectly influence the expression of matrix metalloproteinases (MMPs). Pro-inflammatory cytokines, such as IL-1β and TNF-α, which are upregulated by the inflammasome, can increase the expression of MMPs. This heightened MMP activity contributes to tissue remodeling and mucosal erosion in IBD by degrading extracellular matrix components [59]. Similarly, NLRP3, by producing IL-1β along with TNF-α, and LPS, can upregulate intercellular adhesion molecule 1 (ICAM-1), further perpetuating the inflammatory response [60]. The binding of leukocytes to ICAM1 is a key step that allows these cells to move over the endothelial barrier and invade the *lamina propria* [61,62]. The persistent inflammatory response, supported by ICAM1-mediated leukocyte infiltration, contributes to the impairment of the intestinal barrier, resulting in mucosal erosion and ulceration, which are characteristics of IBD. The involvement of IL-1β as part of the NLRP3 inflammasome highlights its critical role in the inflammation observed in this disease. Network analysis predicts that in addition to NLRP3 and TLRs, proteins like APP, ELAVL1, RELA, and others directly interacting with NLRP3, IL-1β, TLR4, ORMDL3 may also be significant contributors to the IBD pathogenesis. Understanding these interactions can provide deeper insights into the complex inflammatory networks in IBD and open up new avenues for therapeutic intervention.

The generation of reactive oxygen species (ROS) has a significant effect on the development of various inflammatory disorders, including IBD. Produced by polymorphonuclear neutrophils, macrophages, and intestinal epithelial cells, ROS can serve as both signaling molecules and inflammatory promoters [63]. ROS compromises cellular homeostasis by damaging essential macromolecules, leading to cellular injury, and increased mucosal barrier permeability [64]. Additionally, oxidative stress is a significant contributor to gut dysbiosis, leading to the decrease of beneficial anaerobic bacteria and inducing alterations in the microbial diversity in the gut, thereby creating a vicious cycle of damage and inflammation [65]. Although oxidative stress in IBD is influenced by factors such as chronic inflammation, gut dysbiosis, and intestinal epithelial cell stress, it is crucial to emphasize the central role of mitochondrial dysfunction in this context.

Mitochondrial dysfunction significantly exacerbates oxidative stress, as mitochondria are primary ROS sources during cellular respiration [66]. Impaired mitochondrial function not only enhances ROS production but also perpetuates inflammation by activating key inflammatory pathways, such as the NLRP3 inflammasome [67], leading to increased release of pro-inflammatory cytokines and further disrupting intestinal barrier integrity. One of the primary contributors to mitochondrial dysfunction in various chronic diseases is peroxisome proliferator-activated receptor-γ coactivator (PGC-1α) downregulation. PGC-1α plays a crucial role in maintaining mitochondrial health by promoting mitochondrial biogenesis and enhancing the expression of genes involved in oxidative phosphorylation [68] (Figure 3). Upon activation, PGC-1α stimulates the transcription factors NRF1 and NRF2, which are key to mitochondrial biogenesis and function. Along with NRF1, PGC-1α increases the expression of mitochondrial transcription factor A (TFAM), a crucial protein necessary for the replication and transcription of mitochondrial DNA. This TFAM upregulation, combined with the effects of NRF1 and NRF2, leads to the creation of new mitochondria and improves their functionality [69]. In IBD, chronic inflammation, LPS, and cytokine production, particularly TNF-α and IL1-β, are believed to contribute to the downregulation of PGC-1α. These cytokines are found to increase the NF-κB subunit p65 (p65), which interacts with and inactivates PGC-1α, leading to a reduction in its transcriptional activity [70]. This impairment can result in decreased cellular energy production, increased oxidative stress, and a heightened inflammatory response. Network analysis revealed that PGC-1α, a key regulator of oxidative stress and mitochondrial biogenesis, interacts with ORMDL3 via RELA. This suggests that ORMDL3 plays a significant role in these processes and may represent a potential therapeutic target (Figure 3).

Autophagy is one of the most crucial cellular pathways in IBD due to its fundamental role in maintaining cellular homeostasis and immune regulation. This process involves the degradation and recycling of damaged organelles, proteins, and pathogens within cells [71]. Autophagy impairment in IBD results from the complex interplay of genetic mutations, inflammatory cytokines, microbial factors, ERS, oxidative stress, mitochondrial dysfunction, nutrient imbalances, and cellular damage. Consequently, this leads to the accumulation of damaged organelles, proteins, and bacteria, which in turn exacerbates inflammation and contributes to disease pathology. The interaction between autophagy and ER stress is a crucial mechanism that contributes to the breakdown of the intestinal barrier in IBD [72]. Persistent ER stress can inhibit autophagy by disrupting the machinery needed for the autophagic process. This can lead to the accumulation of damaged components, exacerbate epithelial cell dysfunction, and increase permeability, allowing harmful substances to cross the epithelial layer and contribute to inflammation and disease progression in IBD (Figure 1).

As discussed above, ER stress through UPR leads to an expression of ATF6 in intestinal epithelial cells [73]. ATF6, as part of the UPR, can enhance this response by increasing the expression of autophagy-related genes, thus promoting autophagy and aiming to restore cellular homeostasis. When autophagy is insufficient or dysregulated, misfolded proteins and damaged organelles accumulate, leading to cellular dysfunction and contributing to the inflammatory environment characteristic of IBD. On the other hand, the other two UPR branches, eIF2α and PERK, promote the selective translation of ATF4, which results in the upregulation of genes involved in autophagy, especially under conditions of ER stress [74]. According to this, autophagy is critically involved in IBD, particularly in the context of ER stress. Both ATF6 and ATF4 interact with ORMDL3 in our network analysis (Figure 3), suggesting a potential mutual involvement in the disease process. ORMDL3 overexpression can exacerbate ERS, which may lead to heightened activation of ATF6 and ATF4. Dysregulated activation of these transcription factors can result in altered expression of genes responsible for protein folding and degradation. This disruption in cellular homeostasis could further compromise intestinal epithelial integrity and amplify the inflammatory response. Consequently, since ORMDL3 interacts with both ATF6 and ATF4, it again suggests that this gene could be a potential target for therapeutic interventions in IBD.

**Figure 4:**
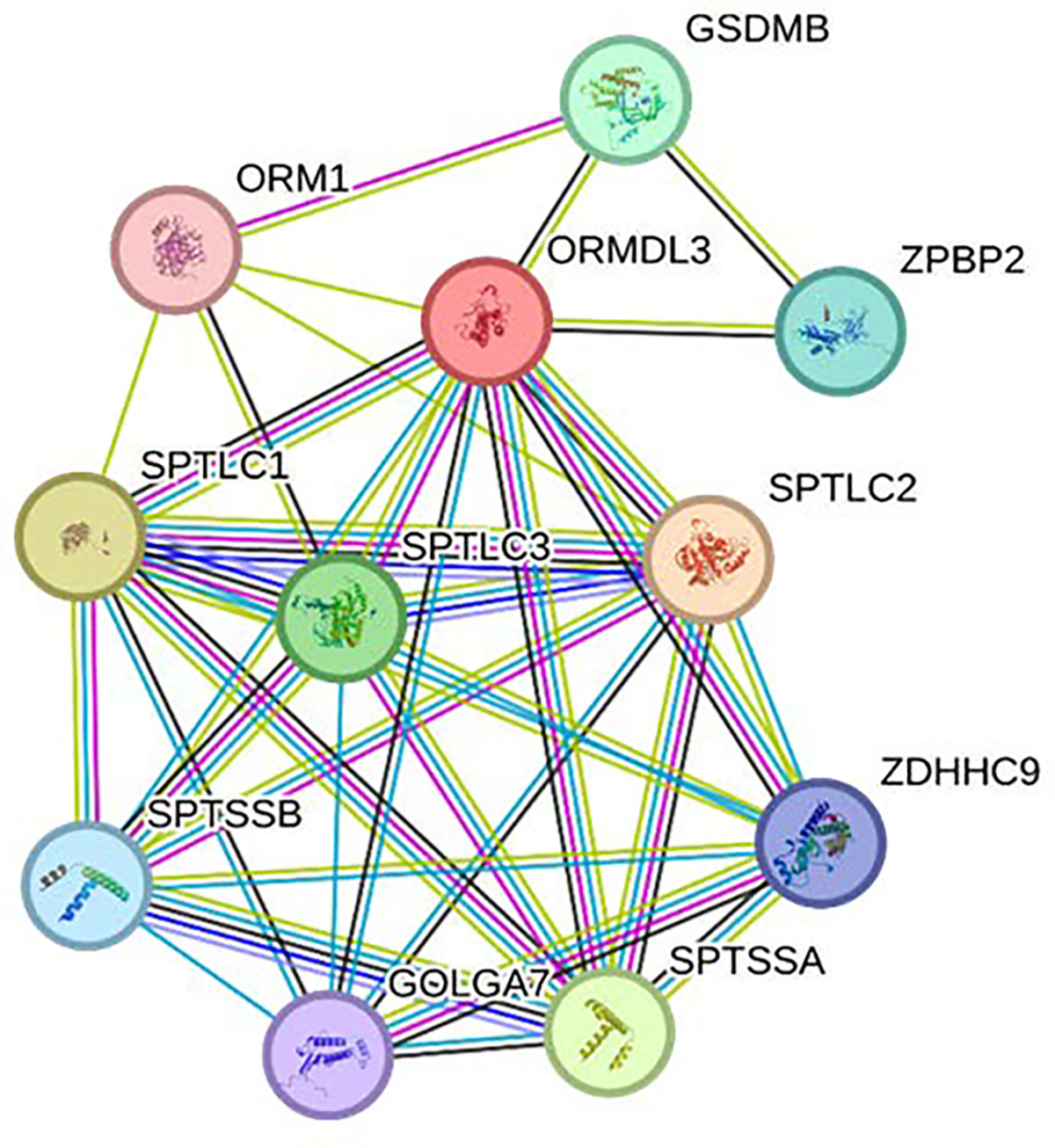
Search Tool for the Retrieval of Interacting Genes/Proteins (STRING) network of ORMDL3. For abbreviation of various proteins/genes, please see the description in the text.

Following the findings from the IMEx database that identified interactions of ORMDL3, we expanded our analysis by using the Search Tool for the Retrieval of Interacting Genes/Proteins (STRING) database to further investigate ORMDL3 interactions and its role in the context of IBD. STRING network revealed an association of ORMDL3 with various serine palmitoyl transferases (SPTs), including SPTLC1, SPTCL3, SPTSSB, and SPTSSA, whose association was also revealed in IMEx analysis (Figure 4). Additionally, STRING network also revealed the association of ORMDL3 with Gasdermin-B (GSDMB), whose N-terminal moiety is known to promote pyroptosis. Since pyroptosis plays a critical role in IBD pathogenesis [75,76,77], ORMDL3 may be a therapeutic target in IBD. Upon activation, the N-terminal moiety of GSDMB forms pores in the cell membrane, which leads to rapid cell swelling, lysis, and the subsequent release of intracellular pro-inflammatory contents, including cytokines like IL-1β and IL-18. These inflammatory mediators amplify the immune response, creating a feedback loop that exacerbates inflammation, leading to the destruction of intestinal epithelial cells, further compromising the gut barrier, and driving chronic inflammation - a hallmark of IBD.

### Clinical Relevance of Targeting ORMDL3 in IBD

As mentioned earlier, ER stress and the UPR play critical roles in the pathogenesis of IBD and signs of ER stress in human ileal and colonic mucosa in CD and UC have been reported [78]. Targeting these pathways holds the potential to reduce inflammation, autophagy, and oxidative stress, which are key contributors to disease progression [37,79]. Despite their importance, current therapeutic options specifically targeting ER stress and UPR in clinical practice are limited. Several in *vivo* and in *vitro* studies have shown that quercetin, Kira6, and 4-phenylbutyric acid (4-PBA) can target ER stress effectively in models of induced colitis [80,81,82]. However, further preclinical and clinical studies are necessary to fully prove their therapeutic potential in treating human IBD. For instance, 4-PBA, an FDA-approved drug, has demonstrated effectiveness in treating rat colitis by significantly reducing ER stress. However, despite its safety profile and anti-colitic activity, the need for very high doses to achieve therapeutic effects in the gut may limit its practicality in clinical settings [83]. Similarly, preclinical and animal studies have recognized tauroursodeoxycholic acid (TUDCA) for its ability to reduce ER stress and its noteworthy efficacy in managing IBD [84]. A recent phase I clinical trial by Huang et al. reported that oral TUDCA led to significant clinical improvements in patients with moderate to severe UC. The treatment successfully reduced ER stress in colonic epithelial cells, resulting in mucosal healing and decreased ER stress markers in some patients [85]. Considering that phase I trials primarily focus on safety and dosage rather than establishing definitive efficacy, further phase II and III trials are essential for comprehensively evaluating the therapeutic potential of TUDCA. These subsequent trials will be crucial in evaluating its effectiveness, optimal dosing, and long-term safety in a larger and more diverse population of IBD patients. Since ER stress and UPR interact with ORMDL3, targeting ORMDL3 and designing novel therapeutics will be of importance.

Activation of NF-κB is a key event in the inflammatory response, leading to the recruitment and activation of immune cells that perpetuate the inflammatory cycle in the intestinal mucosa, exacerbating tissue damage and disease progression. Increased NF-κB expression in human patients and the role of non-canonical NF-κB pathway in CD and UC pathogenesis as well as the role of non-canonical NF-κB signaling as a therapeutic target and biomarker in IBD patients suggest its importance in clinical settings [86,87]. In IBD, NF-κB activation is driven by several stimuli, including bacterial LPS, TLRs (especially TLR4), NLRs, ROS, and pro-inflammatory cytokines [88]. Importantly, ER stress and the UPR, specifically through the IRE1 and PERK pathways (Figure 2), also significantly influence NF-κB activation, linking ER stress to the inflammatory response in IBD and exacerbating chronic inflammation. Numerous clinical trials have shown that infliximab and other TNF-α inhibitors effectively reduce NF-κB activation and improve outcomes in IBD [89,90]. Additionally, tofacitinib, approved for treating UC, has demonstrated efficacy by targeting cytokines involved in NF-κB activation [91]. By inhibiting JAKs, tofacitinib reduces the production and activity of pro-inflammatory cytokines such as IL-6, IL-12, and IL-23, which are known to be involved in the activation of NF-κB [35]. While current treatments indirectly affect NF-κB, direct targeting of this pathway remains under investigation and may lead to more precise and effective IBD therapies in the future.

There are limited options for drugs specifically targeting autophagy and oxidative stress in IBD. Most current treatments for this disease focus on managing inflammation and immune responses rather than directly targeting autophagy or oxidative stress. ORMDL3, involved in ER stress, UPR activation, inflammation, oxidative stress, and autophagy, could be a promising therapeutic target for IBD. Its role in modulating ER stress and UPR pathways is crucial, given their significance in the pathogenesis of this disease. By targeting ORMDL3, it may be possible to mitigate ER stress, reduce chronic inflammation, alleviate oxidative stress, and regulate autophagy, addressing multiple key processes involved in IBD. Current treatments indirectly impact these pathways, but ORMDL3 presents an opportunity for more precise and effective therapies, offering a potential advance in managing IBD through a multifaceted approach.

## Conclusion

IBD continues to pose significant challenges despite the wide range of available therapies and medications. The persistent difficulty in achieving complete healing underscores the need for novel therapeutic approaches. Network analysis identified ORMDL3 as a key player interacting with various factors contributing to inflammation, mitochondrial dysfunction, and autophagy. This highlights the potential of ORMDL3 as a therapeutic target to offer more effective ways to manage IBD, potentially leading to improved patient outcomes and moving closer to the goal of achieving long-term remission or even a cure. The literature review in this article and network analysis suggests ORMDL3 as a potential therapeutic target, however, in-depth research on the role of ORMDL3 in IBD and designing novel molecules to target ORMDL3 should be the focus of future investigations.

## Key points, Outstanding Questions and Challenges

- **Need for novel therapies**: Current treatments for IBD, including anti-inflammatory drugs, immunosuppressants, and biologics, do not provide a definitive cure and often have limited efficacy in some patients.
- **Network analysis:** The network analysis revealed a direct interaction between ORMDL3, TLR2, TLR4, TLR9, HMGB1, IL1β, IL18, ATF4, ATF6, and PGC-1α, suggesting a probable role of ORMDL3 in the pathogenesis of IBD.
- **Underlying mechanisms:** How exactly does ORMDL3 modulate inflammation, mitochondrial dysfunction, and autophagy in the context of IBD?
- **ORMDL3 as a biomarker:** Could ORMDL3 or its downstream effects serve as reliable biomarkers for disease activity, progression, or response to therapy in IBD?
- **Therapeutic potential:** Targeting ORMDL3 could potentially offer a novel approach to improve treatment outcomes and clinical management of IBD.
- **Clinical trial design**: Designing clinical trials that accurately assess the efficacy and safety of ORMDL3-targeted therapies involves addressing complex endpoints and outcome measures.
